# Characterizing immune activation in HIV-exposed but uninfected infants in the first year of life

**DOI:** 10.3389/fimmu.2026.1914801

**Published:** 2026-09-03

**Authors:** Isabelle Roseto, Lori Silveira, Kaili Curtis, Jennifer Canniff, Adriana Weinberg, Christiana Smith

**Affiliations:** 1 School of Medicine, University of Colorado, Aurora, CO, United States; 2 Department of Pediatrics, University of Colorado, Aurora, CO, United States; 3 Departments of Medicine and Pathology, University of Colorado, Aurora, CO, United States

**Keywords:** HIV-exposed uninfected infant, immune activation, immune imprinting, maternal inflammation, perinatal HIV exposure

## Abstract

**Introduction:**

HIV-exposed uninfected (HEU) infants experience multiple health complications compared to HIV-unexposed (HUU) infants, including immune dysregulation in the first year of life. *In utero* exposure to maternal inflammation associated with HIV infection is a proposed mechanism of HEU immune dysregulation. This study compared markers of inflammation and immune activation over the first year of life between HEU and HUU infants.

**Methods:**

Stored plasma samples were sourced from HEU infants enrolled in the International Maternal Pediatric Adolescent AIDS Clinical Trials (IMPAACT) protocol P1025, a prospective observational study that enrolled US pregnant people with HIV and their infants between 2002-2013, and from 3 studies that enrolled healthy HUU infants in Aurora, CO. We used the MesoScale Discovery platform to measure 16 inflammatory and regulatory biomarkers at weeks 2, 6, 24 and 48 of life.

**Results:**

In a multivariable regression analysis of 135 HEU and 51 HUU infants adjusted for sex, race/ethnicity, delivery mechanism, gestational age and birth weight, we identified higher concentrations of several biomarkers in HUU compared to HEU infants, including: IL-17A, IL-1β, IL-4, TNFα, IL-5, IL-8, MCP1, CCL22, CCL3, and CCL4. Among HEU infants, maternal CD4+ T-lymphocyte count of <200 or 200–500 copies/mL at delivery was associated with higher infant concentrations of IFNγ, IL-6, CXCL10, TNFα, CCL3, and CCL4 and maternal viral load ≥400 RNA copies/ml at delivery was associated with higher infant IL-2 and IL-10.

**Conclusion:**

We identified that HUU infants had higher concentrations of most immune biomarkers compared to HEU infants, in contrast to prior literature. We also observed that lower maternal CD4+ T-lymphocyte count and higher HIV viral load at delivery were associated with higher concentrations of several inflammatory biomarkers in HEU infants. This suggests that more advanced maternal HIV may be associated with heightened immune activation in HEU infants that persists into late infancy. These data suggest that there may be multiple subtypes of immune alterations in HEU infants, potentially existing on a spectrum spanning from immune activation to a regulatory or suppressed phenotype. Further studies are needed to determine the mechanisms through which *in utero* exposure to maternal HIV infection impacts infant immune development.

## 1 Introduction

The rate of vertical HIV transmission has declined to less than 1% in most regions of the world (1–4). As a result, approximately 3300 HIV-exposed, but uninfected (HEU) infants are born in the US each year, and >1 million worldwide. Compared to HIV-unexposed (HUU) infants, HEU infants experience myriad health complications, including impacts on growth and neurodevelopment, and infection-related morbidity and mortality during the first two years of life (5–9). HEU infants also demonstrate multifaceted immune and metabolic abnormalities which may underlie their increased clinical pathology (10, 11). Although the majority of clinical data for HEU infants derive from low and middle-income countries (LMIC), several studies have demonstrated high rates of morbidity among HEU versus HUU infants born in high-income countries as well (12–15).

The mechanisms driving the clinical outcomes observed in HEU infants have not been clearly defined. Mounting data demonstrate that *in utero* exposure to inflammatory stimuli can imprint the developing fetus (16–20). HIV infection is characterized by a state of chronic inflammation and immune activation that persists despite suppression of viral replication with antiretroviral therapy (ART) (21–23). Pregnant people living with HIV (PPLHIV) have evidence of increased inflammation and gut microbial translocation compared to pregnant people without HIV, and greater inflammation during pregnancy is associated with more severe immunologic dysregulation and increased morbidity and mortality in HEU infants (24–40). These data suggest that HIV-associated inflammation during pregnancy impacts infant outcomes in a dose-dependent fashion.

Several studies have measured concentrations of inflammatory and regulatory biomarkers in PPLHIV and HEU infants compared to populations unaffected by HIV. Among PPLHIV, a phenotype of immune activation is frequently described, with increased concentrations of IL-1β, IL-6, TNFα, CXCL10 (IP-10), sCD14, and sCD163 as the most consistently reported findings (32–34, 41–44). In cord blood, several studies have reported increased pro-inflammatory cytokines and chemokines in HEU infants, including IL-6, IL-8, TNFα, and CXCL10 (IP-10), whereas findings for T lymphocyte-associated cytokines (e.g. IFNγ, IL-2, IL-17A) and regulatory cytokines (IL-10), are inconsistent (10, 30, 34, 36, 41, 42, 44–47).

Important limitations constrain the interpretation of these studies. First, the majority utilize cord blood as a proxy for infant samples. However, emerging evidence has demonstrated that cord blood immune profiles may be biased by perinatal factors and the acute inflammatory response associated with the stress of birth (34, 48, 49). Additionally, most of these studies rely on cross-sectional sampling at delivery, which limits our understanding of how immune alterations evolve over time. Only a small number of studies include measurements beyond the neonatal period, and even fewer have followed infants at multiple timepoints throughout the first year of life (44, 45, 50, 51). This gap is particularly significant given that the increased clinical morbidity observed in HEU infants has been reported up to 2 years of age. The overall trajectory and duration of immune dysregulation in HEU infants beyond the neonatal period remain incompletely defined.

To address these gaps, we conducted a retrospective study to characterize inflammatory profiles in HEU infants at multiple time points across the first year of life and compare those to HUU infants. We also examined the relationship between maternal HIV disease severity near delivery and infant inflammatory biomarker concentrations.

## 2 Materials and methods

### 2.1 Plasma sample collection

HEU samples were sourced from infants enrolled in the International Maternal Pediatric Adolescent AIDS Clinical Trials (IMPAACT) protocol P1025, a prospective observational study that enrolled pregnant people with HIV and their infants between 2002–2013 in the US (52). P1025 collected serial peripheral blood samples from infants during the first year of life (53). Sample collection windows for the HEU infants were based on infant age as follows: 2 weeks (+1 week), 6 weeks (+2 weeks), 16 weeks (+2 weeks), 24 weeks (+4 weeks), 48 weeks (+/-12 weeks) (54). All enrolled PPLIV received ART according to standard of care at the time of enrollment. HEU infants received zidovudine postnatal prophylaxis in accordance with standard of care in the United States during the time that the parent study (IMPAACT P1025) was conducted. HUU plasma samples were sourced from 3 separate studies that enrolled healthy pregnant people and/or infants in Aurora, CO and collected infant samples between 2016-2022. Sample collection windows for HUU infants were based on infant age as follows: 2 weeks (+/-1 week), 6 weeks (+/- 2 weeks), 24 weeks (+6 weeks), 48 weeks (+/- 8 weeks).

Parents or guardians who signed consent for these studies agreed to banking of their infants’ samples for future research. The use of archived samples for this study was approved by the Colorado Multiple Institutional Review Board (protocol #24-0655).

### 2.2 Detection of plasma biomarkers

Frozen plasma aliquots were thawed and used in batched analyses. Biomarkers were measured in duplicate using custom multiplex electrochemiluminesence microarrays according to the manufacturer’s instructions (Meso Scale Diagnostics, Rockville, MD). The custom panels included proinflammatory cytokines (interleukin [IL]-1β, IL-6, TNFα); proinflammatory chemokines (CXCL8 [IL-8], CXCL10 [IP-10], monocyte chemoattractant protein [MCP1; CCL2], macrophage-derived chemokine [MDC; CCL22]); markers of T-cell differentiation (interferon [IFN]-γ, IL-2, IL-4, IL-5, IL-12p70, IL-17A); markers of macrophage activation (macrophage inflammatory protein [MIP-1α; CCL3], MIP-1β, [CCL4]); and regulatory cytokines (IL-10).

### 2.3 Statistical analysis

Statistical analyses were performed in SAS 9.4 or Rstudio version 2025.05.1. *A priori* power analyses determined that including a minimum of 72 infants in the HEU group and 20 in the HUU group at each time point provided a power of ≥0.85 to detect significant differences. Demographic and clinical characteristics were compared between HEU and HUU infants using Pearson’s chi-square or Fisher’s exact tests, or Wilcoxon rank sum tests, as appropriate. The HEU cohort included a single set of twins; twin B was selected for exclusion from all analyses.

We performed four independent comparisons of biomarker concentrations between HEU vs. HUU infants at each of the timepoints (2, 6, 24, and 48 weeks of life). This comparison was not conducted at week 16 because HUU samples were not available for that timepoint. For biomarker concentrations below the limit of detection, we used half of the lower limit of detection. No biomarker concentrations were above the upper limit of quantification. We tested the plasma biomarker concentrations for normality using Shapiro-Wilks tests in the two groups and then log_2_ transformed the data to make the biomarkers compatible with parametric analyses. Univariable and multivariable analyses were performed on these log-transformed data. The univariable analyses were performed using linear models with log_2_ transformed biomarker concentration as the dependent variable and group = HEU vs. HUU as the independent variable. The multivariable analyses adjusted for infant sex, race/ethnicity, delivery mode, gestational age at delivery, and birthweight, if these variables were significant (p<0.05) in the univariable analyses. P-values were adjusted for multiple comparisons using the Benjamini Hochberg procedure to control the false discovery rate. We also constructed a principal component analysis (PCA) to reduce the data dimensionality. We constructed Principal Components 1 & 2 from the loadings and the standardized log2 transformed biomarker concentrations for each subject and then examined the differences between the mean values of the components between the HEU and HUU infants using Student’s t-tests.

Among HEU infants, we evaluated for an association between maternal plasma HIV RNA and/or CD4 T-lymphocyte levels closest to the time of delivery and the concentrations of infant biomarkers. Maternal “closest to delivery” laboratory values were chosen from results available between 28 weeks’ gestation and the date of delivery. We stratified the HIV RNA and CD4 values into groups (CD4 <200, 200-500, >500 cells/µL; HIV RNA <400, ≥400 copies/mL) and used general linear model ANOVAs to evaluate if differences between the groups existed. Finally, we compared biomarker concentrations between HEU infants in the “most severe” maternal HIV categories (i.e. CD4 <200 or HIV RNA ≥400) vs. HUU infants using univariable linear models.

## 3 Results

We included samples from 135 HEU and 51 HUU infants (**Table 1**). HEU and HUU infants were relatively well matched for sex, but HEU infants had lower birth weights and gestational age at delivery and were more likely to be born via cesarean section. There were also differences in race and ethnicity, with more Black non-Hispanic HEU infants and more White non-Hispanic HUU infants. People with HIV in the US were discouraged from breastfeeding during the years of P1025 enrollment; therefore, none of the HEU infants breastfed, but the majority of HUU infants were exposed to at least some breast milk. In the HEU cohort, 17% of infants were admitted to the hospital within the first year of life, 48% of whom were admitted for infectious causes. Hospitalization data are not available for the HUU cohort.

**Table 1:** Demographics and clinical characteristics of participants.

Participant Characteristics: Median (IQR) or Count (%)	Level	HEU	HUU	P-value
**n**		**135**	**51**	
Sex (%)	Female	61 (45.2)	27 (52.9)	0.411
	Male	74 (54.8)	24 (47.1)	
Birth Weight (grams)		3044 (2800-3325)	3220 (2928-3510)	0.019
Race/Ethnicity (%)	Asian non-Hispanic	0 (0.0)	3 (5.9)	<0.001
	Black non-Hispanic	66 (48.9)	5 (9.8)	
	Hispanic	52 (38.5)	17 (33.3)	
	White non-Hispanic	13 (9.6)	21 (41.2)	
	Other/Unknown	4 (3.0)	5 (9.8)	
Delivery Mode (%)	Cesarean section	66 (48.9)	15 (29.4)	0.014
	Vaginal	69 (51.1)	35 (68.6)	
	Unknown	0 (0.0)	1 (2.0)	
Gestational Age at Delivery (weeks)		38 (37, 39)	39 (38.5-40)	<0.001
Breastfed (%)	Exclusive until 6 months	0 (0.0)	15 (29.4)	<0.001
	Some breastfeeding	0 (0.0)	33 (64.7)	
	Not breastfed	135 (100.0)	3 (5.9)	
Birth Year		2004 (2003-2009)	2022 (2016-2022)	<0.001
Pregnancy Outcome (%)	Singleton	134 (99.3)	51 (100.0)	1.000
	Twin	1 (0.7)	0 (0.0)	
Infant hospitalizations within the first year of life (%)		23 (17.0)	*	-
Infant hospitalizations due to infection, out of total hospitalizations (%)		11 (48.8)	*	–
Final antiretroviral regimen received prior to delivery (%)	None	1 (0.7)	–	–
	2 NRTI + MVC	1 (0.7)	–	–
	2 NRTI + NNRTI	23 (17.0)	–	–
	2 NRTI + PI	91 (67.4)	–	–
	2 NRTI + PI + INSTI	3 (2.2)	–	–
	2 NRTI + PI + NNRTI	3 (2.2)	–	–
	NNRTI + PI + INSTI	1 (0.7)	–	–
	NRTI only	13 (9.6)	–	–
Maternal CD4 count at entry (cells/µL)		395 (268-618)	–	–
Maternal CD4 count at delivery (cells/µL)		511 (353-688)	–	–
Maternal HIV viral load at entry (copies/mL)		2949 (100-17,600)	–	–
Maternal HIV viral load at delivery (copies/mL)		50 (50-400)	–	–
Maternal HIV viral load <400 copies/mL at delivery (%)		87 (68.0%)	–	–

HEU, HIV-exposed uninfected; HUU, HIV-unexposed uninfected; IQR, interquartile range; INSTI, integrase inhibitor; MVC, maraviroc; NRTI, nucleos(t)ide reverse transcriptase inhibitor; NNRTI, non-nucleoside reverse transcriptase inhibitor; PI, protease inhibitor.

*Information not available.

With the exception of 1 participant who received no ART during pregnancy, mothers living with HIV received antiretroviral treatment according to the standard of care in the United States at the time that the parent study (IMPAACT P1025) was conducted. Among 136 women with HIV, 43 (31.6%) initiated ART prior to conception of the pregnancy. The remaining 92 women received ART for a median (IQR) of 21 (16.6-24.4) weeks prior to delivery. The antiretroviral regimen received often changed during pregnancy. The final antiretroviral regimen received prior to delivery is listed in **Table 1**, with most women (91, 66.9%) receiving a protease inhibitor-based final regimen. Maternal median (IQR) CD4+ T-lymphocyte count was 395 (268-618) cells/µL at entry and 511 (353-688) cells/µL at delivery, and maternal plasma HIV RNA was 2949 (100-17,600) copies/mL at entry and 50 (50-400) copies/mL at delivery.

We performed univariable and multivariable regression analyses to compare the plasma biomarker concentrations between HEU and HUU infants at 2, 6, 24, and 48 weeks of life. At each timepoint, biomarker concentrations were compared between 72 HEU infants and 20 HUU infants. Among the 135 HEU infants, plasma samples were available at all 4 timepoints for 6 infants, 3 timepoints for 62 infants, 2 timepoints for 8 infants, and 1 timepoint for 59 infants. Among the 51 HUU infants, plasma samples were available at all 4 timepoints for 0 infants, 3 timepoints for 10 infants, 2 timepoints for 8 infants, and 1 timepoint for 33 infants. The distribution of infants contributing plasma samples at each timepoint is shown in **Supplementary Tables 1**, **2**.

**Figure 1:**
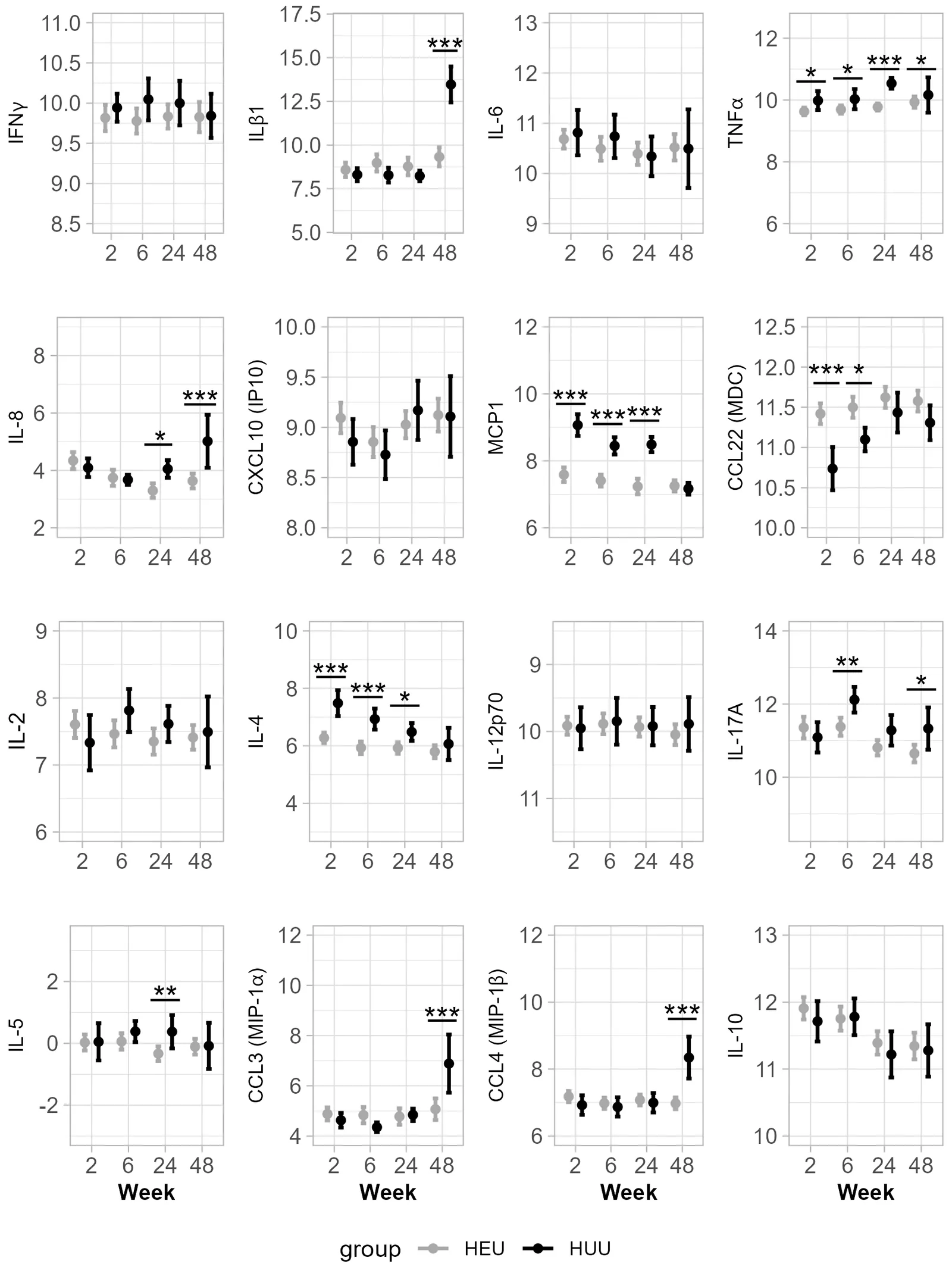
Plasma biomarker concentrations in HIV-exposed uninfected (HEU) and HIV-unexposed uninfected (HUU) infants at weeks 2, 6, 24, and 48 of life. All biomarkers are presented as log2-transformed concentrations in pg/mL. Points on the graphs indicate the group mean (95%CI) for HEU (grey) & HUU (black) at each time point. Comparisons between groups at each time point were performed using multivariable logistic regression after adjustment for infant sex, race/ethnicity, delivery mode, gestational age at delivery, and birthweight, and p-values were adjusted for multiple comparisons (*p<0.05, **p<0.01, ***p<0.001). Total N included at each time point was HEU n=72; HUU n=20. HEU, HIV-exposed uninfected; HUU, HIV-unexposed uninfected. Dot and whisker plots showing log2-transformed biomarker concentrations among HEU (gray) and HUU (black) infants at weeks 2, 6, 24, and 48 of life.

We identified lower concentrations in HEU compared with HUU plasma of multiple proinflammatory cytokines and chemokines, markers of T-cell differentiation, and macrophage activation (**Figure 1**; **Supplementary Table 3**). Differences were seen at all four timepoints (weeks 2, 6, 24, and 48), although the pattern of divergence or convergence between HEU and HUU biomarker concentrations varied between the biomarkers (e.g. some biomarkers differed more in early infancy, others in later infancy). Differences in Th2 and macrophage activation tended to occur more often in later infancy, whereas differences in proinflammatory cytokines and chemokines and T-cell activation occurred throughout the first year of life. The sole biomarker that was found at higher concentrations in HEU compared to HUU plasma was CCL22 (MDC) at weeks 2 and 6. (**Figure 1**; **Supplementary Table 3**).

**Figure 2:**
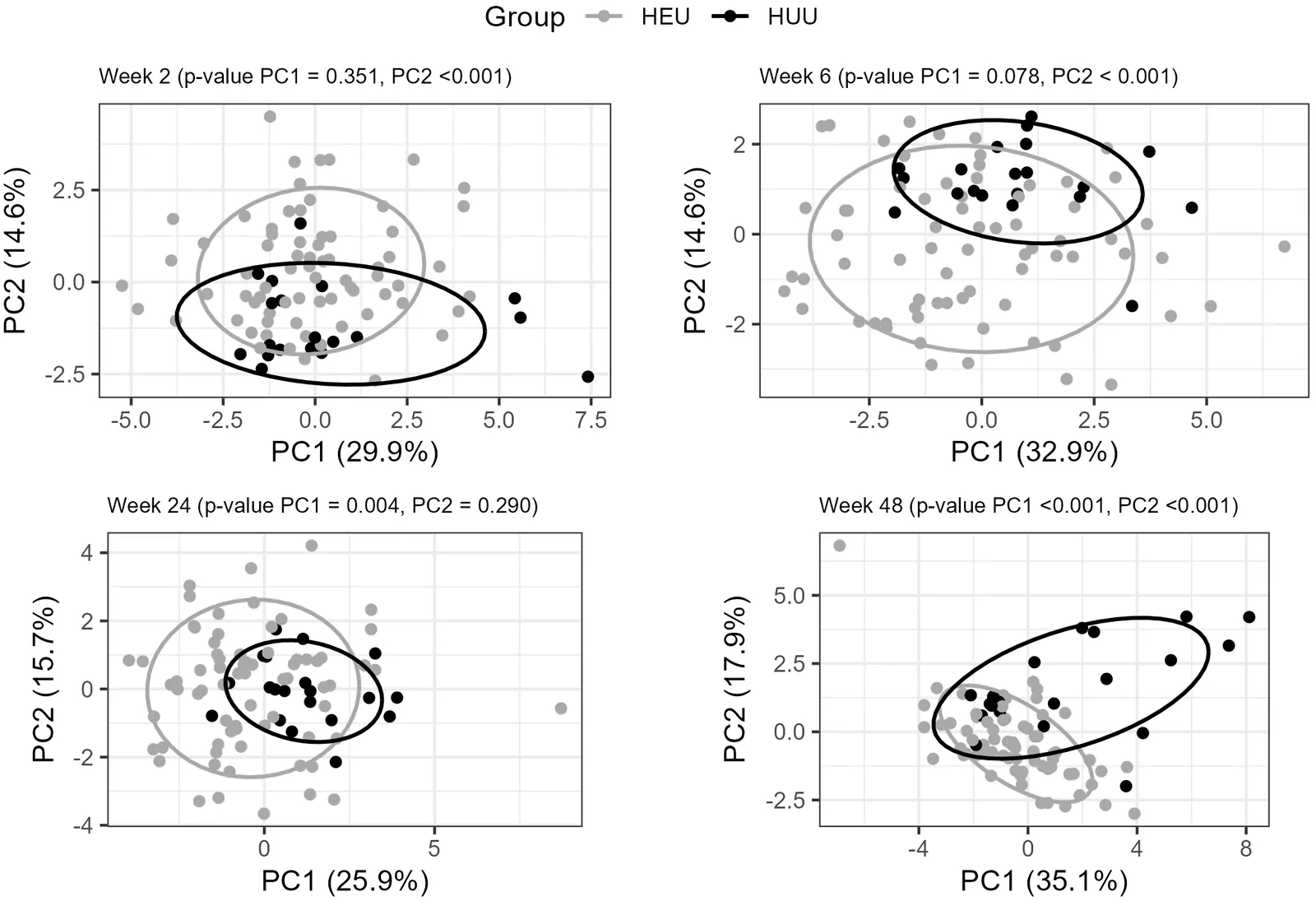
Bioprofile relatedness of plasma biomarkers in HEU and HUU infants over the first year of life. Principal component analysis (PCA) of plasma biomarker concentrations at weeks 2, 6, 24, and 48 of life. Each point represents an individual infant. Gray points indicate HEU infants and black points indicate HUU infants. Ellipses represent 95% confidence intervals for each group. The percentage of variance explained by principal components 1 (PC1) and 2 (PC2) is indicated on the axes. Four-panel principal component analysis (PCA) plots showing plasma biomarker profiles in HEU (gray) and HUU (black) infants at Weeks 2, 6, 24, and 48. Each point represents one infant, with ellipses indicating group clustering.

Principal component analysis (PCA) was performed at each timepoint to assess patterns of variance across the measured biomarkers (**Figure 2****;** **Supplementary Table 4**). Across all timepoints, PC1 was primarily driven by pro-inflammatory cytokines and chemokines and markers of T-cell differentiation, including IFNγ, IL-2, IL-4, IL-6, IL-12p70, CXCL10 (IP-10), and TNFα. PC2 captured a pattern of variance characterized by chemokines and select cytokines, including IL-8, MCP1, CCL22 (MDC), and CCL4 (MIP1β). CCL22 (MDC) contributed strongly to PC2 at weeks 2 and 6, corresponding to the timepoints in which the concentration of CCL22 in HEU infants was significantly higher than HUU. Together, these findings suggest that a profile of specific biomarkers distinguish HEU from HUU infants during the first year of life.

**Figure 3:**
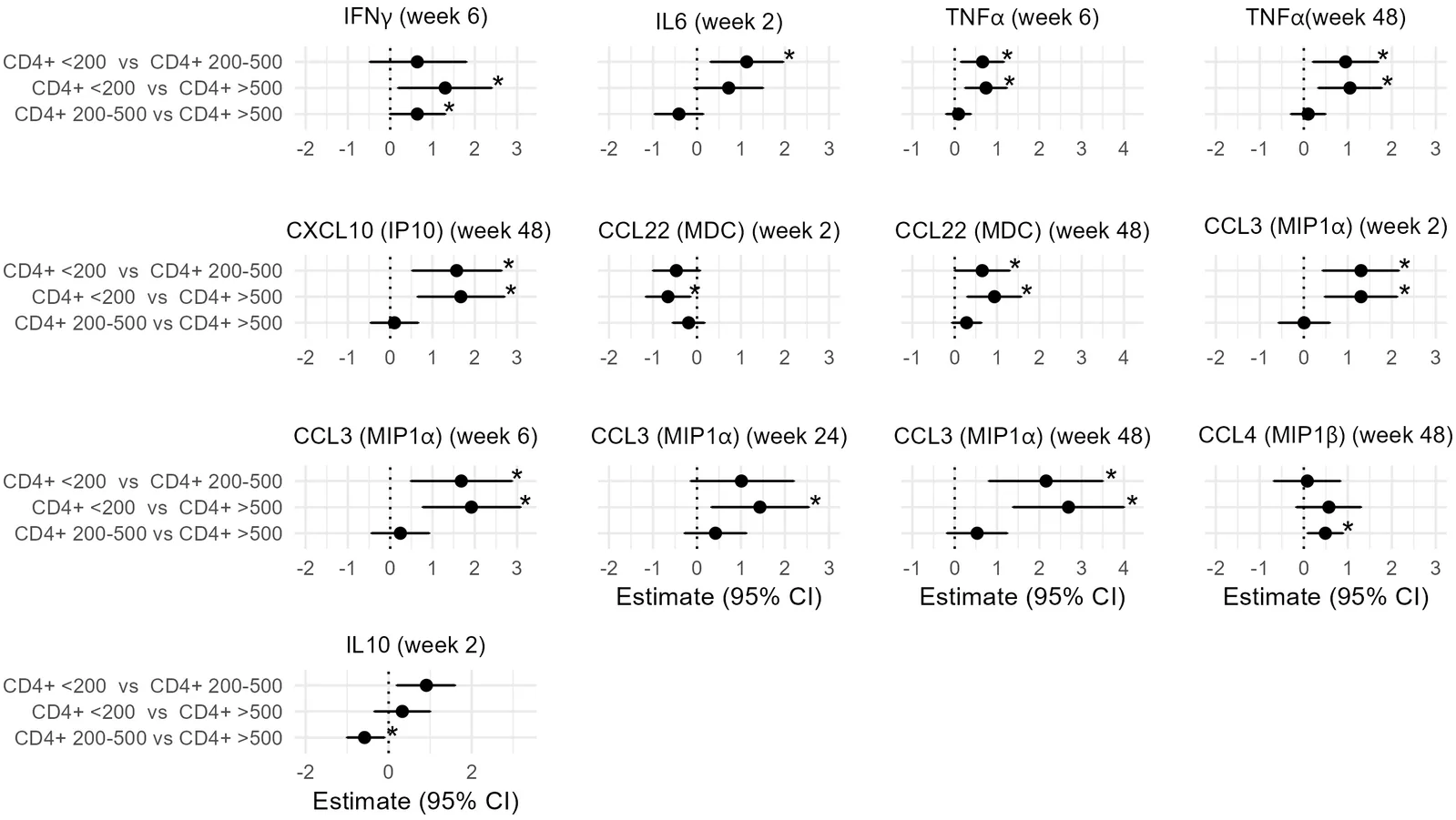
Relationship of maternal CD4+ T-lymphocyte count closest to delivery with HIV-exposed infant plasma biomarker concentrations over the first year of life. Forest plots are shown for the plasma biomarkers whose concentrations differed significantly between infants whose mothers had CD4 counts of <200, 200-500, and >500 cells/µL. Biomarker comparisons show pairwise effect estimates and 95% confidence intervals from unadjusted general linear model ANOVA analyses. Statistical significance is indicated by asterisks (*p<0.05, **p<0.01, ***p<0.001). Total N included at each time point was n=72 HEU infants. CCL, chemokine C-C motif ligand; CXCL, chemokine C-X-C motif ligand; IFN, interferon; IL, interleukin; MDC, macrophage derived chemokine; MIP, macrophage inflammatory protein; TNF, tumor necrosis factor. Forest plots showing differences in biomarker concentrations between infants whose mothers had CD4 counts of <200, 200-500, and >500 cells/µL.

To explore whether maternal HIV disease severity influenced the HEU immune biomarker profile, we examined infant biomarker concentrations according to maternal CD4+ T lymphocyte count and maternal HIV viral load closest to delivery (**Figure 3**; **Supplementary Tables 5**, **6**). Maternal CD4 counts had an inverse relationship with the concentrations of multiple infant inflammatory cytokines and chemokines and markers of macrophage activation. In unadjusted analyses, infants born to mothers with CD4 counts <200 or 200–500 cells/µl, compared to mothers with higher CD4 counts, had higher concentrations of IFNγ at week 6, IL-6 at week 2, TNFα at weeks 6 and 48, CXCL10 (IP10) at week 48, CCL3 (MIP1α) at all four timepoints, and CCL4 (MIP1β) at week 48. IL-10, a regulatory cytokine, had a direct relationship with maternal CD4 at 2 weeks. CCL22 (MDC) had a direct relationship with maternal CD4 at 2 weeks, but an inverse relationship with maternal CD4 at 48 weeks. The majority of these associations did not retain statistical significance after adjusting for multiple comparisons. Similarly, detectable maternal HIV viral load (HIV RNA ≥400 copies/mL) was directly associated with concentrations of select biomarkers in HEU infants. Infants born to mothers with higher viral loads had increased IL-10 concentrations at weeks 6 and 48 and increased IL-2 concentrations at week 48, compared to infants born to mothers with suppressed viral load. Again, these findings did not retain statistical significance after adjustment for multiple comparisons.

To further understand the bimodal effect of *in utero* HIV exposure on infant cytokine and chemokine plasma concentrations, we compared select biomarker concentrations among HEU infants exposed to advanced and/or poorly controlled HIV infection (i.e. CD4 <200 or HIV RNA ≥400) to those of HUU infants. Many biomarker levels were higher in HEU infants born to mothers with advanced HIV than in HUU infants, but the majority of these comparisons did not demonstrate statistical significance.

## 4 Discussion

We characterized systemic inflammatory profiles in HEU compared to HUU infants across the first year of life. Surprisingly, we observed lower concentrations of multiple biomarkers of inflammation and immune activation in HEU infants, including IL-1β, IL-4, IL-5, IL-8, IL-17A, MCP1, CCL3 (MIP-1α), CCL4 (MIP-1β) and TNFα. These differences were seen at all four time points examined, suggesting a sustained alteration in immune signaling over the first year of life. The clinical implications of a generally hypo-inflammatory immune signature in HEU infants remain uncertain. Although excess inflammation has been shown to be harmful, insufficient inflammatory responses may impair effective pathogen clearance (55–58). Lower concentrations of inflammatory cytokines and chemokines necessary for the recruitment and activation of neutrophils, monocytes/macrophages, and T-cells (e.g. IL-8, MCP1, CCL3 [MIP1α], CCL4 [MIP1β]) may contribute to the increased susceptibility to bacterial and respiratory infections reported in this population.

Despite the overall reduction in inflammatory mediators, we identified increased concentrations of CCL22 (MDC) in HEU compared to HUU infants at weeks 2 and 6 of life. CCL22 (MDC) is a chemokine produced by innate immune cells such as macrophages and dendritic cells, which plays an important role in regulating the trafficking of T-cell subsets, especially Th2 and regulatory T cells (59–61). Therefore, increased levels of CCL22 (MDC) may indicate increased immune regulation or tolerogenic signaling. Prior studies have demonstrated that HEU infants exhibit increased frequencies of regulatory T cells that correlate with decreased effector T cell function, supporting the concept of a regulatory or tolerogenic immune phenotype in this population (62). Increased CCL22 (MDC) concentrations are also associated with chronic infection and have been described in advanced HIV infection (63, 64). Furthermore, we have previously demonstrated higher concentrations of CCL22 (MDC) in pregnant people with versus without HIV, which potentially could result in increased placental transfer of CCL22 (MDC) to HEU infants (34). This hypothesis aligns with our finding of the increased concentrations of CCL22 (MDC) in early infancy that diminish over the first year of life.

Among HEU infants, we observed that markers of increased maternal HIV disease severity were associated with higher concentrations of several inflammatory and regulatory biomarkers in unadjusted analyses, including IFNγ, IL-2, IL-6, IL-10, TNFα, CXCL10 (IP-10), CCL3 (MIP1α), and CCL4 (MIP1β). This bimodal effect of *in utero* exposure to HIV suggests a complex interaction between the maternal and fetal immune systems. *In utero* exposure to severe maternal immune suppression (evidenced by CD4 T-cell counts <200 cells/µL and/or uncontrolled viral replication) appears to be associated with a nonspecific increase in inflammatory biomarker secretion. However, in the absence of conditions that lead to this nonspecific increase, we observe a downregulatory effect of *in utero* exposure to maternal HIV on the infant immune system.

Most prior publications describing inflammatory biomarkers in HEU infants have identified higher concentrations in HEU infants, particularly in cord blood and early infancy (30, 41, 42). However, not all publications have been uniform, with some studies identifying lower concentrations of IFNγ, IL-4, IL-5, IL-10, and IL-12p70 in HEU compared to HUU infant cord blood (34, 42, 46, 47). As described above, cord blood is greatly influenced by perinatal stress which can trigger vigorous inflammatory responses in the fetus (34, 48, 49). The underlying inflammatory state of the neonate at birth (whether activated or suppressed) may be masked by this perinatal inflammatory response.

Three recent studies evaluated plasma biomarker concentrations outside of the neonatal period, with Evans et al. measuring plasma biomarkers at 1 month of age, and Yin et al. and Prinsloo et al. up to 6 months of age (44, 50, 51). Both Evans et al. and Yin et al. reported generally increased inflammatory biomarker concentrations in HEU compared to HUU infants. Similar to our study, Yin et al. identified that the immune dysregulation observed in HEU infants persisted to 6 months of age; however, the specific biomarkers whose concentrations differed between HEU and HUU infants were not consistent over time. In contrast, Prinsloo et al. reported that HEU infants demonstrated lower concentrations of inflammatory biomarkers like IFNγ and CRP and higher concentrations of the anti-inflammatory biomarker TGFβ, although the majority of these comparisons did not reach statistical significance. Differences in maternal health and environmental exposures, timing of sample collection, biomarkers measured, and recruitment time period may contribute to the variation in inflammatory profiles observed between these different cohorts.

This study has notable strengths, including the inclusion of HEU samples from multiple timepoints throughout the first year of life, and inclusion of participants from a high-income country, an understudied population in HEU immunology research. To our knowledge, this is one of the few studies to examine inflammatory markers beyond six months of age in HEU infants. Additionally, we avoided the use of cord blood plasma, which can be biased by the stress of birth.

This study also has several limitations to consider. First, HUU and HEU samples were derived from different cohorts and had an 18-year difference in median birth years, which are potential confounding variables. Also, the HEU cohort is geographically broad, and was recruited from multiple sites within the US, whereas the HUU cohort was only recruited from one site (Aurora, CO, USA). However, we adjusted for differences in demographic and clinical factors to the extent possible in the multivariable analyses. We were unable to adjust for breast milk exposure, which differed significantly between HEU and HUU cohorts. Breastfeeding has well-established effects on infant immune maturation and may have contributed in part to the differences identified between HEU and HUU infants (65, 66). We were not able to include samples from the same HEU and HUU infants at all timepoints. Particularly within the HUU cohort, the findings should be interpreted as repeated cross-sectional analyses. However, the consistency of findings across multiple time points supports the robustness of our results. The use of archived plasma samples is dependent upon adequate sample handling and storage; however, all samples were handled and stored according to strict protocols and underwent fewer than 3 freeze-thaw cycles, and these conditions have previously been shown to have a minimal effect on cytokine concentration (61, 62). Though the storage duration differed between the two groups, prior studies have shown that when stored appropriately, long term storage has minimal effect on cytokine concentration (67). Lastly, plasma biomarkers only provide a partial view of immune function and may not fully reflect tissue-level immunity.

In conclusion, we identified differences in plasma cytokine and chemokine concentrations between HEU and HUU infants that persist to at least 48 weeks of life. These findings may provide novel insight into the duration and nature of immune alterations in HEU infants from a high-income country setting. Within this cohort, we found that HEU infants’ immune profiles were characterized by broadly reduced pro-inflammatory cytokines and selectively increased CCL22 (MDC) in early infancy, in comparison to HUU infants. However, HEU infants exposed to more advanced maternal HIV were more likely to demonstrate a phenotype of immune activation in unadjusted analyses. These findings suggest that *in utero* exposure to maternal HIV and its associated inflammatory state may result in long-lasting immune imprinting, possibly manifesting as two different subsets of responses: suppressed vs. activated. Further mechanistic studies integrating functional immune assays are needed to confirm how these two distinct patterns of immune alterations might contribute to the increased risk of infectious morbidity observed in HEU infants.

## Data Availability

The original contributions presented in the study are included in the article/**Supplementary Material**. Further inquiries can be directed to the corresponding author.
