# Supplementary material for: Characterizing immune activation in HIV-exposed but uninfected infants in the first year of life
Source: Front Immunol. 2026 Sep 3;17:1914801. doi: 10.3389/fimmu.2026.1914801 (PMC13582223; doi:10.3389/fimmu.2026.1914801)
Supplement: Supplementary file 1 [file SupplementaryFile1.docx]

Supplementary Material

# 1 Supplementary Tables

**Supplemental Table 1.** Distribution of HEU infants according to availability of plasma samples across study timepoints.

| Timepoint (weeks)* | 2 | 6 | 24 | 48 | Number of HEU infants |
| --- | --- | --- | --- | --- | --- |
|  | X | X | X | X | 6 |
|  | X | X | X |  | 31 |
|  | X | X |  | X | 3 |
|  | X |  | X | X | 3 |
|  | X |  | X |  | 2 |
|  | X | X |  |  | 3 |
|  | X |  |  |  | 24 |
|  |  | X | X | X | 26 |
|  |  | X | X |  | 1 |
|  |  | X |  |  | 2 |
|  |  |  | X | X | 2 |
|  |  |  | X |  | 1 |
|  |  |  |  | X | 32 |
| **Total samples** | **72** | **72** | **72** | **72** |  |

*Each row represents a unique pattern of sample availability. “X” indicates that a plasma sample was used at that timepoint.

**Supplemental Table 2.** Distribution of HUU infants according to availability of plasma samples across study timepoints.

| Timepoint (weeks)* | 2 | 6 | 24 | 48 | Number of HUU infants |
| --- | --- | --- | --- | --- | --- |
|  | X | X | X |  | 10 |
|  | X | X |  |  | 6 |
|  | X |  |  |  | 3 |
|  |  | X | X |  | 2 |
|  |  | X |  |  | 2 |
|  |  |  | X |  | 8 |
|  |  |  |  | X | 20 |
| **Total samples** | **19** | **20** | **20** | **20** |  |

*Each row represents a unique pattern of sample availability. “X” indicates that a plasma sample was used at that timepoint.

**Supplemental Table 3**. Univariable and multivariable analyses of plasma inflammatory biomarkers in HEU and HUU infants

|  |  | **Univariable Analysis – geometric mean (95%CI)** | | | | | | **Multivariable Analysis*** | | | | | | |
| --- | --- | --- | --- | --- | --- | --- | --- | --- | --- | --- | --- | --- | --- | --- |
| Cytokine | Time Point (week) | HEU | HUU | p-value | | Adjusted  p-value | | HEU | | HUU | | p-value | | Adjusted p-value |
| Proinflammatory cytokines | | | | | | | | | | | | | | |
| IL-1β | 2 | 341 (259, 448) | 316 (187, 533) | 0.801 | 0.879 | |  | |  | |  | |  | |
|  | 6 | 480 (350, 659) | 310 (173, 557) | 0.201 | 0.444 | |  | |  | |  | |  | |
|  | 24 | 432 (314, 549) | 301 (166, 545) | 0.293 | 0.521 | |  | |  | |  | |  | |
|  | 48 | 593 (402, 877) | 19177 (9221, 39881) | **<0.001** | **<0.001** | | 706 (457, 1091) | | 13976 (6588, 29651) | | **<0.001** | | **<0.001** | |
| IL-6 | 2 | 1766 (1497, 2084) | 1799 (1315, 2461) | 0.920 | 0.950 | |  | |  | |  | |  | |
|  | 6 | 1413 (1177, 1696) | 1607 (1168,2180) | 0.519 | 0.755 | |  | |  | |  | |  | |
|  | 24 | 1290 (1065, 1563) | 1147 (803, 1636) | 0.568 | 0.760 | |  | |  | |  | |  | |
|  | 48 | 1583 (1197, 2093) | 2184 (1295, 3684) | 0.289 | 0.521 | |  | |  | |  | |  | |

| TNFα | 2 | 793 (724, 869) | 1011 (897, 1141) | **0.017** | 0.060 | 793 (724, 869) | 1011 (897, 1141) | **0.017** | **0.020** |
| --- | --- | --- | --- | --- | --- | --- | --- | --- | --- |
|  | 6 | 829 (751, 916) | 1045 (870, 1255) | **0.033** | 0.106 | 829 (751, 916) | 1045 (870, 1255) | **0.033** | **0.036** |
|  | 24 | 878 (813, 949) | 1487 (1288, 1718) | **<0.001** | **<0.001** | 878 (813, 949) | 1487 (1288, 1718) | **<0.001** | **<0.001** |
|  | 48 | 977 (799, 1195) | 2173 (1491, 3168) | **0.034** | 0.105 | 977 (799, 1195) | 2173 (1491, 3168) | **0.034** | **0.037** |
| Proinflammatory chemokines | | | | | | | | | |
| IL-8 | 2 | 20 (17, 26) | 17 (12, 25) | 0.415 | 0.648 |  |  |  |  |
|  | 6 | 12 (10, 15) | 13 (9, 18) | 0.853 | 0.895 |  |  |  |  |
|  | 24 | 8 (7, 10) | 17 (12, 23) | **<0.001** | 0.004 | 8 (7, 10) | 17 (12, 23) | **0.001** | **0.016** |
|  | 48 | 9 (7, 12) | 81 (48, 136) | **<0.001** | <0.001 | 9 (7, 12) | 81 (48, 136) | **<0.001** | **<0.001** |
| CXCL10 (IP-10) | 2 | 614 (518, 726) | 662 (481, 912) | 0.680 | 0.821 |  |  |  |  |
|  | 6 | 518 (442, 606) | 399 (298, 533) | 0.126 | 0.299 |  |  |  |  |
|  | 24 | 580 (490, 686) | 778 (570, 1062) | 0.105 | 0.258 |  |  |  |  |
|  | 48 | 843 (691, 1027) | 662 (457, 960) | 0.263 | 0.497 |  |  |  |  |
| Monocyte chemoattractant protein | | | | | | | | | |
| MCP1 | 2 | 187 (161, 216) | 537 (406, 709) | **<0.001** | **<0.001** | 211 (176, 252) | 482 (355, 654) | **<0.001** | **<0.001** |
|  | 6 | 165 (147, 186) | 349 (279, 436) | **<0.001** | **<0.001** | 169 (147, 195) | 330 (260, 420) | **<0.001** | **<0.001** |
|  | 24 | 134 (115, 157) | 359 (270, 478) | **<0.001** | **<0.001** | 144 (122, 170) | 302 (223, 410) | **<0.001** | **<0.001** |
|  | 48 | 132 (116, 150) | 131 (103, 167) | 0.976 | 0.976 |  |  |  |  |
| Macrophage-derived chemokine | | | | | | | | | |
| CCL22 (MDC) | 2 | 2883 (2597, 3202) | 1705 (1397, 2081) | **<0.001** | **<0.001** | 2870 (2524, 3264) | 1740 (1395, 2169) | **<0.001** | **<0.001** |
|  | 6 | 3177 (2885, 3498) | 2193 (1833, 2624) | **<0.001** | **0.002** | 3144 (2804, 3525) | 2226 (1877, 2760) | **0.009** | **0.012** |
|  | 24 | 3623 (3256, 4031) | 2913 (2391, 3548) | 0.060 | 0.167 |  |  |  |  |
|  | 48 | 3453 (3086, 3864) | 2816 (2282, 3476) | 0.096 | 0.246 |  |  |  |  |

| Markers of T-cell differentiation | | | | | | | | | |
| --- | --- | --- | --- | --- | --- | --- | --- | --- | --- |
| IFNγ | 2 | 1166 (962, 1414) | 1479 (1026, 2130) | 0.264 | 0.496 |  |  |  |  |
|  | 6 | 1217 (991, 1494) | 1466 (1004, 2142) | 0.399 | 0.638 |  |  |  |  |
|  | 24 | 1178 (982, 1414) | 1272 (907, 1785) | 0.697 | 0.826 |  |  |  |  |
|  | 48 | 1502 (1164, 1937) | 1279 (794, 2061) | 0.562 | 0.760 |  |  |  |  |
| IL-2 | 2 | 159 (131, 192) | 194 (135, 280) | 0.338 | 0.585 |  |  |  |  |
|  | 6 | 163 (134, 198) | 242 (167, 349) | 0.063 | 0.168 |  |  |  |  |
|  | 24 | 138 (106, 164) | 184 (129, 264) | 0.137 | 0.313 |  |  |  |  |
|  | 48 | 138 (107, 178) | 238 (147, 386) | 0.053 | 0.154 |  |  |  |  |
| IL-4 | 2 | 78 (68, 88) | 179 (139, 232) | **<0.001** | <0.001 | 78 (68, 88) | 179 (139, 232) | **<0.001** | **<0.001** |
|  | 6 | 59 (50, 70) | 122 (90, 165) | **<0.001** | <0.001 | 59 (50, 70) | 122 (90, 165) | **<0.001** | **<0.001** |
|  | 24 | 62 (54, 70) | 89 (70, 115) | **0.010** | 0.038 | 62 (54, 70) | 89 (70, 115) | **0.010** | **0.012** |
|  | 48 | 58 (48, 69) | 67 (48, 94) | 0.448 | 0.667 |  |  |  |  |
| IL-5 | 2 | 1.02 (0.83, 1.26) | 1.03 (0.69, 1.54) | 0.949 | 0.964 |  |  |  |  |
|  | 6 | 1.03 (0.87, 1.23) | 1.30 (0.95, 1.8) | 0.214 | 0.457 |  |  |  |  |
|  | 24 | 0.76 (0.62, 0.94) | 1.49 (1.01, 2.2) | **0.004** | **0.017** | 0.76 (0.62, 0.94) | 1.49 (1.01, 2.2) | **0.004** | **0.006** |
|  | 48 | 0.84 (0.65, 1.08) | 0.94 (0.58, 1.53) | 0.679 | 0.821 |  |  |  |  |
| IL-12p70 | 2 | 982 (886, 1088) | 935 (770, 1136) | 0.665 | 0.821 |  |  |  |  |
|  | 6 | 952 (835, 1084) | 989 (777, 1258) | 0.785 | 0.879 |  |  |  |  |
|  | 24 | 1034 (925, 1155) | 969 (789, 1190) | 0.590 | 0.760 |  |  |  |  |
|  | 48 | 960 (774, 1191) | 1087 (727, 1627) | 0.594 | 0.760 |  |  |  |  |
| IL-17A | 2 | 2329 (1836, 2955) | 2179 (1388, 3421) | 0.799 | 0.879 |  |  |  |  |
|  | 6 | 2656 (2225, 3170) | 4448 (3208, 6167) | **0.008** | **0.032** | 2518 (2075, 3057) | 4596 (3293, 6416) | **0.005** | **0.007** |
|  | 24 | 1680 (1438, 1962) | 2492 (1863, 3332) | **0.020** | 0.067 | 1723 (1445, 2054) | 2521 (1814, 3505) | 0.066 | 0.067 |
|  | 48 | 1531 (1267, 1849) | 2367 (1662, 3373) | **0.035** | 0.107 | 1465 (1193, 1800) | 2308 (1617, 3293) | **0.037** | **0.040** |
| Markers of macrophage activation | | | | | | | | | |
|  | 2 | 132 (117, 149) | 122 (96, 153) | 0.532 | 0.757 |  |  |  |  |
| CCL3 (MIP-1α) | 2 | 27 (23, 32) | 23 (17, 32) | 0.440 | 0.667 |  |  |  |  |
|  | 6 | 23 (19, 28) | 19 (13, 27) | 0.352 | 0.593 |  |  |  |  |
|  | 24 | 21 (18, 26) | 28 (19, 40) | 0.226 | 0.467 |  |  |  |  |
|  | 48 | 25 (19, 33) | 87 (52, 146) | **<0.001** | **0.005** | 23 (17, 31) | 99 (60, 163) | **<0.001** | **<0.001** |
| CCL4 (MIP-1β) | 6 | 111 (99, 125) | 109 (87, 135) | 0.842 | 0.895 |  |  |  |  |
|  | 24 | 118 (104, 134) | 123 (97, 155) | 0.786 | 0.879 |  |  |  |  |
|  | 48 | 105 (90, 122) | 325 (244, 434) | **<0.001** | **<0.001** | 105 (90, 122) | 325 (244, 434) | **<0.001** | **<0.001** |
| Regulatory cytokines | | | | | | | | | |
| IL-10 | 2 | 4798 (4070, 5655) | 4114 (3009, 5624) | 0.396 | 0.638 |  |  |  |  |
|  | 6 | 4055 (3489, 4714) | 3713 (2805, 4914) | 0.586 | 0.760 |  |  |  |  |
|  | 24 | 3116 (2659, 3653) | 2563 (1908, 3441) | 0.254 | 0.496 |  |  |  |  |
|  | 48 | 2371 (1908, 2946) | 2509 (1671, 3766) | 0.810 | 0.879 |  |  |  |  |

Abbreviations: HEU, HIV-exposed uninfected; HUU, HIV-unexposed uninfected; CI, confidence interval; IL, interleukin; IFNγ, interferon-gamma; TNFα, tumor necrosis factor-alpha; MCP1, monocyte chemoattractant protein-1; MDC, macrophage-derived chemokine; IP-10, interferon-γ–induced protein 10.

**Supplemental Table 4**. Principal component loadings for inflammatory biomarkers at weeks 2, 6, 24, and 48 of life

|  | Weights PC1 | PC2 | PC1 | PC2 | PC1 | PC2 | PC1 | PC2 |
| --- | --- | --- | --- | --- | --- | --- | --- | --- |
| Variable | Wk2 | Wk2 | Wk6 | Wk6 | Wk24 | Wk24 | WK48 | WK48 |
| IFNγ | 0.34 |  | 0.33 |  | 0.38 |  | 0.23 | -0.35 |
| IL-10 | 0.32 |  | 0.32 |  | 0.29 | 0.25 | 0.23 | -0.35 |
| IL-12p70 | 0.30 |  | 0.31 |  | 0.22 |  | 0.24 | -0.26 |
| IL-17A |  | -0.26 | 0.20 | 0.33 |  | 0.22 |  | 0.40 |
| IL-1β |  | 0.29 |  | -0.46 |  | -0.44 | 0.20 |  |
| IL-2 | 0.31 | -0.31 | 0.34 | 0.29 | 0.36 | 0.29 | 0.34 |  |
| IL-4 | 0.22 |  | 0.31 |  | 0.26 |  | 0.21 |  |
| IL-6 | 0.24 |  | 0.29 |  | 0.29 |  | 0.30 |  |
| TNFα | 0.38 |  | 0.36 |  | 0.35 |  | 0.37 |  |
| IL-5 | 0.28 |  | 0.29 |  |  | -0.33 | 0.22 |  |
| IL-8 |  | 0.45 |  | -0.25 |  |  | 0.27 | 0.41 |
| CXCL10 (IP-10) | 0.34 |  | 0.27 | -0.25 | 0.36 |  | 0.23 | -0.29 |
| MCP1 |  | -.35 |  | 0.42 | 0.28 |  |  |  |
| CCL22 (MDC) |  | 0.44 |  | -0.33 |  | -0.41 |  |  |
| CCL3 (MIP-1α) | 0.21 | 0.21 | 0.20 |  |  |  | 0.28 |  |
| CCL4 (MIP-1β) |  | 0.37 |  | -0.22 |  | -0.47 | 0.30 | 0.31 |

Abbreviations: PC, principal component; Wk, week of life; IL, interleukin; IFNγ, interferon-gamma; TNFα, tumor necrosis factor-alpha; MCP1, monocyte chemoattractant protein-1; MDC, macrophage-derived chemokine; IP-10, interferon-γ–induced protein 10.

**Supplemental Table 5**. Geometric mean (95%CI) inflammatory biomarker concentration (pg/mL) in HEU infants by maternal CD4+ T-lymphocyte count closest to delivery

|  | Week | Maternal CD4  <200 cells/ml  n=12 | Maternal CD4  200-500 cells/ml  n=45 | Maternal CD4  >500 cells/ml  n=66 | p-value | adjusted p-value |
| --- | --- | --- | --- | --- | --- | --- |
| Proinflammatory cytokines | | | | | | |
| IL-1β | 2 | 183 (71, 474) | 361 (228, 572) | 388 (252, 596) | 0.248 | 0.588 |
|  | 6 | 885 (312, 2507) | 499 (291, 858) | 471 (278, 800) | 0.609 | 0.812 |
|  | 24 | 451 (90, 2276) | 447 (209, 955) | 413 (260, 657) | 0.987 | 0.996 |
|  | 48 | 519 (170, 1582) | 542 (242, 1214) | 636 (379, 1067) | 0.920 | 0.981 |
| IL-6 | 2 | 3126 (1280, 7636)* | 1424 (1008, 2012) | 1893 (1573, 2280) | **0.028** | 0.192 |
|  | 6 | 2653 (973, 7230) | 1203 (881, 1644) | 1492 (1139, 1954) | 0.095 | 0.338 |
|  | 24 | 1073 (694, 1660) | 1087 (843, 1401) | 1325 (955, 1839) | 0.600 | 0.812 |
|  | 48 | 2625 (915, 7530) | 1225 (868, 1728) | 1698 (1158, 2489) | 0.237 | 0.583 |
| TNFα | 2 | 896 (576, 1395) | 762 (629, 923) | 815 (724, 917) | 0.597 | 0.812 |
|  | 6 | 1309 (794, 2157)*^Ω^ | 830 (696, 989) | 783 (696, 881) | **0.015** | 0.137 |
|  | 24 | 1058 (732, 1530) | 907 (786, 1047) | 808 (728, 898) | 0.107 | 0.352 |
|  | 48 | 1830 (941, 3558) | 944 (798, 1118) | 883 (727, 1074) | **0.018** | 0.144 |
| Proinflammatory chemokines | | | | | | |
| IL-8 | 2 | 15 (8, 30) | 19 (14, 27) | 22 (15, 32) | 0.522 | 0.745 |
|  | 6 | 18 (7, 49) | 12 (9, 18) | 12 (8, 16) | 0.506 | 0.745 |
|  | 24 | 8 (3, 17) | 10 (8, 13) | 7 (5, 9) | 0.112 | 0.352 |
|  | 48 | 11 (3, 45) | 10 (7, 15) | 8 (6, 11) | 0.436 | 0.715 |
| CXCL10 (IP-10) | 2 | 794 (340, 1858) | 530 (395, 710) | 615 (503, 752) | 0.335 | 0.670 |
|  | 6 | 898 (283, 2850) | 547 (391, 763) | 449 (373, 540) | 0.082 | 0.309 |
|  | 24 | 481 (291, 797) | 603 (461, 788) | 507 (412, 624) | 0.502 | 0.745 |
|  | 48 | 2295 (933, 5647)*^Ω^ | 771 (564, 1054) | 719 (562, 920) | **0.007** | 0.112 |
| Monocyte chemoattractant protein | | | | | | |
| MCP1 | 2 | 266 (124, 572) | 175 (133, 231) | 176 (141, 221) | 0.268 | 0.591 |
|  | 6 | 167 (76, 368) | 164 (128, 209) | 165 (140, 194) | 0.996 | 0.996 |
|  | 24 | 118 (61, 228) | 136 (98, 189) | 123 (102, 149) | 0.813 | 0.913 |
|  | 48 | 110 (50, 242) | 133 (102, 173) | 126 (105, 153) | 0.796 | 0.913 |
| Macrophage-derived chemokine | | | | | | |
| CCL22 (MDC) | 2 | 2026 (1452, 2826)^Ω^ | 2805 (2272, 3464) | 3175 (2705, 3726) | **0.045** | 0.221 |
|  | 6 | 3305 (2580, 4233) | 3325 (2767, 3996) | 3043 (2578, 3590) | 0.738 | 0.908 |
|  | 24 | 3992 (2598, 6134) | 3822 (3066, 4764) | 3324 (2877, 3841) | 0.423 | 0.715 |
|  | 48 | 5671 (2746, 11713)^Ω^ | 3608 (3064, 4249) | 2965 (2522, 3485) | **0.011** | 0.137 |
| Markers of T-cell differentiation | | | | | | |
| IFNγ | 2 | 2171 (1256, 3752) | 1084 (757, 1551) | 1202 (937, 1541) | 0.071 | 0.307 |
|  | 6 | 2405 (541, 10698) | 1540 (1025, 2314) | 994 (799, 1236) | **0.030** | 0.192 |
|  | 24 | 1144 (567, 2308) | 1332 (919, 1931) | 1001 (822, 1220) | 0.331 | 0.670 |
|  | 48 | 3802 (435, 33255) | 1555 (1019, 2373) | 1285 (894, 1848) | 0.131 | 0.365 |
| IL-2 | 2 | 180 (112, 288) | 129 (84, 198) | 170 (130, 221) | 0.414 | 0.715 |
|  | 6 | 246 (65, 922) | 169 (117, 244) | 145 (109, 195) | 0.410 | 0.715 |
|  | 24 | 136 (76, 244) | 140 (102, 191) | 119 (85, 166) | 0.765 | 0.910 |
|  | 48 | 191 (64, 571) | 153 (105, 221) | 121 (83, 177) | 0.524 | 0.745 |
| IL-4 | 2 | 79 (49, 126) | 74 (57, 95) | 82 (68, 99) | 0.768 | 0.910 |
|  | 6 | 68 (28, 163) | 61 (46, 81) | 58 (44, 75) | 0.872 | 0.962 |
|  | 24 | 65 (41, 103) | 58 (45, 75) | 61 (49, 76) | 0.888 | 0.963 |
|  | 48 | 83 (49, 141) | 58 (44, 76) | 53 (41, 70) | 0.458 | 0.732 |
| IL-5 | 2 | 1.63 (0.66, 4.02) | 1 (0.63, 1.59) | 0.96 (0.71, 1.29) | 0.351 | 0.681 |
|  | 6 | 1.04 (0.69, 1.58) | 1.1 (0.74, 1.63) | 1.02 (0.8, 1.32) | 0.950 | 0.981 |
|  | 24 | 1.2 (0.5, 2.89) | 0.61 (0.47, 0.79) | 0.82 (0.58, 1.16) | 0.171 | 0.438 |
|  | 48 | 1.17 (0.62, 2.18) | 0.86 (0.51, 1.46) | 0.81 (0.56, 1.18) | 0.805 | 0.913 |
| IL-12p70 | 2 | 1230 (1042, 1452) | 928 (759, 1133) | 1019 (876, 1185) | 0.259 | 0.591 |
|  | 6 | 1336 (780, 2287) | 1049 (825, 1334) | 857 (718, 1022) | 0.116 | 0.352 |
|  | 24 | 1208 (948, 1539) | 1010 (829, 1231) | 1001 (831, 1206) | 0.648 | 0.846 |
|  | 48 | 1584 (1151, 2181) | 1008 (808, 1258) | 834 (550, 1264) | 0.367 | 0.691 |
| IL-17A | 2 | 1857 (736, 4687) | 2351 (1434, 3855) | 2903 (2029, 4154) | 0.510 | 0.745 |
|  | 6 | 1782 (676, 4696) | 3328 (2329, 4755) | 2547 (2009, 3229) | 0.170 | 0.438 |
|  | 24 | 2056 (1175, 3596) | 1739 (1356, 2229) | 1649 (1288, 2110) | 0.722 | 0.908 |
|  | 48 | 1995 (577, 6893) | 1554 (1153, 2094) | 1494 (1165, 1917) | 0.726 | 0.908 |
| Markers of macrophage activation | | | | | | |
| CCL3 (MIP-1α) | 2 | 60 (15, 244)*^Ω^ | 25 (19, 32) | 24 (21, 28) | **0.007** | 0.112 |
|  | 6 | 75 (8, 728)*^Ω^ | 23 (17, 31) | 19 (15, 25) | **0.006** | 0.112 |
|  | 24 | 47 (6, 352)^Ω^ | 23 (17, 32) | 17 (14, 22) | **0.037** | 0.197 |
|  | 48 | 122 (8, 1880)*^Ω^ | 27 (18, 42) | 19 (16, 23) | **<0.001** | **<0.001** |
| CCL4 (MIP-1β) | 2 | 91 (59, 140) | 146 (116, 183) | 138 (112, 171) | 0.121 | 0.352 |
|  | 6 | 105 (59, 185) | 113 (90, 141) | 114 (96, 135) | 0.940 | 0.981 |
|  | 24 | 105 (45, 242) | 140 (113, 174) | 102 (87, 119) | 0.081 | 0.309 |
|  | 48 | 128 (52, 314) | 121 (99, 149)^∞^ | 86 (72, 103) | **0.035** | 0.197 |
| Regulatory cytokines | | | | | | |
| IL-10 | 2 | 6995 (4819, 10155) | 3731 (2838, 4904) | 5672 (4572, 7036) | **0.013** | 0.137 |
|  | 6 | 7509 (3086, 18268) | 4243 (3200, 5627) | 3777 (3058, 4665) | 0.072 | 0.307 |
|  | 24 | 4052 (2788, 5889) | 2842 (2167, 3727) | 2977 (2360, 3754) | 0.432 | 0.715 |
|  | 48 | 4504 (1858, 10923) | 2072 (1564, 2745) | 2383 (1597, 3557) | 0.297 | 0.634 |

- *comparison between <200 to 200-500 significant at the p<0.05 level
- ^Ω^comparison between < 200 to >500 significant at the p <0.05 level
- ^∞^comparison between 200-500 to > 500 significant at the p <0.05 level

Abbreviations: HEU, HIV-exposed uninfected; HUU, HIV-unexposed uninfected; CI, confidence interval; IL, interleukin; IFNγ, interferon-gamma; TNFα, tumor necrosis factor-alpha; MCP1, monocyte chemoattractant protein-1; MDC, macrophage-derived chemokine; IP-10, interferon-γ–induced protein 10.

**Supplemental Table 6**. Geometric mean (95%CI) inflammatory biomarker concentration (pg/mL) in HEU infants by maternal viral load closest to delivery

|  | Week | Maternal HIV RNA <400 copies/mL n=86 | Maternal HIV RNA  ≥400 copies/mL  n=41 | p-value | adjusted  p-value |
| --- | --- | --- | --- | --- | --- |
| Proinflammatory cytokines | | | | | |
| IL-1β | 2 | 343 (241, 488) | 349 (178, 683) | 0.965 | 0.980 |
|  | 6 | 487 (314, 755) | 612 (330, 1135) | 0.551 | 0.765 |
|  | 24 | 500 (307, 814) | 337 (181, 627) | 0.319 | 0.619 |
|  | 48 | 757 (441, 1297) | 416 (234, 737) | 0.140 | 0.532 |
| IL-6 | 2 | 1656 (1355, 2024) | 2347 (1532, 3596) | 0.107 | 0.532 |
|  | 6 | 1281 (1015, 1618) | 1899 (1327, 2717) | 0.063 | 0.532 |
|  | 24 | 1295 (963, 1741) | 1221 (905, 1646) | 0.793 | 0.890 |
|  | 48 | 1363 (986, 1885) | 1859 (1268, 2726) | 0.218 | 0.558 |
| TNFα | 2 | 796 (720, 879) | 810 (625, 1050) | 0.874 | 0.917 |
|  | 6 | 786 (700, 882) | 950 (785, 1149) | 0.077 | 0.532 |
|  | 24 | 882 (794, 980) | 858 (730, 1008) | 0.761 | 0.886 |
|  | 48 | 917 (775,1085) | 1034 (834, 1283) | 0.374 | 0.659 |
| Proinflammatory chemokines | | | | | |
| IL-8 | 2 | 19 (15, 24) | 22 (13, 37) | 0.545 | 0.765 |
|  | 6 | 12 (9, 15) | 14 (9, 22) | 0.487 | 0.765 |
|  | 24 | 9 (7, 11) | 7 (5, 9) | 0.114 | 0.532 |
|  | 48 | 9 (7, 12) | 9 (6, 12) | 0.788 | 0.890 |
| CXCL10 (IP-10) | 2 | 640 (536, 764) | 521 (335, 812) | 0.300 | 0.618 |
|  | 6 | 492 (398, 608) | 578 (411, 813) | 0.402 | 0.677 |
|  | 24 | 618 (480, 795) | 466 (387, 561) | 0.122 | 0.532 |
|  | 48 | 683 (541, 864) | 1042 (762, 1424) | 0.029 | 0.496 |
| Monocyte chemoattractant protein | | | | | |
| MCP1 | 2 | 187 (159, 221) | 166 (106, 259) | 0.521 | 0.765 |
|  | 6 | 173 (147, 203) | 148 (117, 189) | 0.287 | 0.618 |
|  | 24 | 144 (113, 184) | 112 (89, 142) | 0.172 | 0.550 |
|  | 48 | 132 (108, 162) | 125 (103, 152) | 0.704 | 0.868 |
| Macrophage-derived chemokine | | | | | |
| CCL22 (MDC) | 2 | 3080 (2705, 3506) | 2453 (1923, 3130) | 0.095 | 0.532 |
|  | 6 | 3156 (2743, 3630) | 3233 (2691, 3883) | 0.842 | 0.916 |
|  | 24 | 3760 (3250, 4351) | 3288 (2731, 3960) | 0.259 | 0.618 |
|  | 48 | 3165 (2751, 3642) | 3666 (2986, 4501) | 0.217 | 0.558 |
| Markers of T-cell differentiation | | | | | |
| IFNγ | 2 | 1090 (882, 1348) | 1616 (1099, 2377) | 0.075 | 0.532 |
|  | 6 | 1161 (890, 1514) | 1436 (954, 2161) | 0.372 | 0.659 |
|  | 24 | 1271 (957, 1687) | 1012 (789, 1297) | 0.279 | 0.618 |
|  | 48 | 1342 (935, 1927) | 1801 (1175, 2761) | 0.295 | 0.618 |
| IL-2 | 2 | 148 (117, 187) | 206 (144, 294) | 0.158 | 0.532 |
|  | 6 | 146 (111, 192) | 196 (134, 286) | 0.216 | 0.558 |
|  | 24 | 139 (103, 186) | 123 (86, 176) | 0.613 | 0.785 |
|  | 48 | 110 (76, 160) | 188 (152, 234) | **0.031** | 0.496 |
| IL-4 | 2 | 76 (65, 88) | 84 (61, 117) | 0.527 | 0.765 |
|  | 6 | 57 (47, 70) | 65 (44, 97) | 0.508 | 0.765 |
|  | 24 | 63 (52, 77) | 58 (47, 72) | 0.580 | 0.773 |
|  | 48 | 53 (42, 67) | 61 (47, 80) | 0.430 | 0.706 |
| IL-5 | 2 | 0.92 (0.71, 1.18) | 1.44 (0.88, 2.37) | 0.095 | 0.532 |
|  | 6 | 0.95 (0.74, 1.23) | 1.29 (0.99, 1.67) | 0.157 | 0.532 |
|  | 24 | 0.74 (0.57, 0.97) | 0.78 (0.53, 1.14) | 0.855 | 0.917 |
|  | 48 | 0.73 (0.53, 1.01) | 1.06 (0.65, 1.73) | 0.183 | 0.558 |
| IL-12p70 | 2 | 982 (877, 1100) | 1022 (805, 1298) | 0.74 | 0.886 |
|  | 6 | 899 (751, 1076) | 1121 (959, 1311) | 0.127 | 0.532 |
|  | 24 | 1043 (892, 1219) | 1022 (833, 1253) | 0.874 | 0.916 |
|  | 48 | 914 (626, 1334) | 1004 (824, 1222) | 0.705 | 0.867 |
| IL-17A | 2 | 2217 (1640, 2999) | 2691 (1299, 5575) | 0.562 | 0.765 |
|  | 6 | 2485 (1948, 3170) | 3003 (2096, 4303) | 0.381 | 0.659 |
|  | 24 | 1565 (1264, 1938) | 1868 (1407, 2481) | 0.315 | 0.618 |
|  | 48 | 1376 (1110, 1705) | 1798 (1296, 2494) | 0.151 | 0.532 |
| Markers of macrophage activation | | | | | |
| CCL3 (MIP-1α) | 2 | 26 (21, 32) | 28 (18, 44) | 0.75 | 0.886 |
|  | 6 | 20 (16, 27) | 32 (20, 51) | 0.072 | 0.532 |
|  | 24 | 20 (15, 27) | 24 (16, 36) | 0.520 | 0.765 |
|  | 48 | 24 (17, 33) | 27 (18, 39) | 0.611 | 0.785 |
| CCL4 (MIP-1β) | 2 | 140 (120, 164) | 111 (89, 137) | 0.134 | 0.532 |
|  | 6 | 113 (97, 131) | 113 (89, 143) | 0.994 | 0.996 |
|  | 24 | 124 (104, 148) | 103 (82, 130) | 0.195 | 0.558 |
|  | 48 | 107 (91, 127) | 92 (73, 116) | 0.265 | 0.618 |
| Regulatory cytokines | | | | | |
| IL-10 | 2 | 4647 (3915, 5516) | 5461 (3919, 7610) | 0.369 | 0.659 |
|  | 6 | 3629 (2965, 4442) | 5444 (4109, 7213) | **0.024** | 0.496 |
|  | 24 | 3090 (2465, 3875) | 3059 (2399, 3900) | 0.953 | 0.980 |
|  | 48 | 1902 (1395, 2593) | 3247 (2241, 4705) | **0.029** | 0.496 |
